# Differences in Physical Activity and Diet Patterns between Non-Rural and Rural Adults

**DOI:** 10.3390/nu10111601

**Published:** 2018-11-01

**Authors:** Lacey McCormack, Howard Wey, Jessica Meendering, Bonny Specker

**Affiliations:** 1 Health & Nutritional Sciences, South Dakota State University, Brookings, SD 57007, USA; jessica.meendering@sdstate.edu; 2 College of Nursing, South Dakota State University, Brookings, SD 57007, USA; howard.wey@sdstate.edu; 3 EA Martin Program in Human Nutrition, South Dakota State University, Brookings, SD 57007, USA; bonny.specker@sdstate.edu

**Keywords:** rural, Hutterite, physical activity, diet, behavior, lifestyle

## Abstract

Background: It is unclear how rural occupations and lifestyles may play a role in shaping physical activity and diet behaviors that contribute to the rural–urban obesity disparity. Methods: Data come from the prospective and observational South Dakota Rural Bone Health Study, which included adults aged 20–66 years in three groups: (1) non-rural non-Hutterite, (2) rural non-Hutterite, and (3) rural Hutterite. Physical activity data were collected using 7-day physical activity questionnaires, and hours per day in physical activity categories are reported. Diet data were collected using food frequency questionnaires, and food group servings per day (svg/day) are reported. Mixed models were generated to determine group differences in physical activity and diet outcomes, and marginal group means are presented. Results: Among females, both rural groups spent more time in moderate activity (4.8 ± 0.13 h/day and 4.7 ± 0.09 h/day vs. 3.5 ± 0.11 h/day, both *p* < 0.001) and vigorous activity (0.58 ± 0.03 h/day and 0.53 ± 0.02 h/day vs. 0.43 ± 0.03 h/day, both *p* < 0.01) and less time sitting (4.4 ± 0.13 h/day and 4.3 ± 0.09 h/day vs. 5.0 ± 0.11 h/day, both *p* < 0.001) on weekdays than non-rural groups. Hutterite females spent fewer hours in moderate activity (2.6 ± 0.08 h/day vs. 4.5 ± 0.11 h/day, *p* < 0.001) and vigorous activity (0.18 ± 0.02 h/day vs. 0.46 ± 0.02 h/day, *p* < 0.001) on weekend days compared to rural females. Hutterite females consumed more fruits (2.2 ± 0.06 svg/day vs. 1.7 ± 0.10 svg/day, *p* < 0.001) and vegetables (3.6 ± 0.08 svg/day vs. 2.7 ± 0.12 svg/day, *p* < 0.001) than rural females. Among males, both rural groups spent more time in moderate activity (4.9 ± 0.13 h/day and 6.1 ± 0.12 h/day vs. 3.0 ± 0.16 h/day, both *p* < 0.001) and less time sitting (4.1 ± 0.13 h/day and 3.4 ± 0.12 h/day vs. 6.0 ± 0.15 h/day, both *p* < 0.001) on weekdays compared to non-rural groups. Hutterite males spent less time in moderate activity (2.1 ± 0.10 h/day vs. 4.1 ± 0.11 h/day, *p* < 0.001) and vigorous activity (0.15 ± 0.04 h/day vs. 0.74 ± 0.04 h/day, *p* < 0.001) on weekend days compared to rural males. Hutterite males consumed more vegetables (3.0 ± 0.10 svg/day vs. 2.0 ± 0.11 svg/day, *p* < 0.001) than rural males. Conclusions: A rural occupation and lifestyle appear to contribute to differences in physical activity, while traditional rural lifestyle practices contribute to differences in diet.

## 1. Introduction

An obesity disparity exists between rural and urban adults [1,2]. Data from the National Health and Nutrition Examination Survey (NHANES) indicate that 39.6% of rural adults and 33.4% of urban adults are obese [1]. While physical activity and diet behaviors contribute to weight status and ultimately the rural–urban obesity disparity, research that examines differences in physical activity and diet patterns in rural versus urban adults is lacking. Further, while rural socioeconomic and environmental factors have been identified as potentially promoting obesity [3], little research has examined how being “rural” (i.e., having a rural occupation or living a rural lifestyle) contributes to physical activity and diet behaviors, hence the need to not only compare rural versus urban populations, but also to assess individuals who reside in similar geographic locations but have different rural lifestyles.

Physical activity has been assessed in a variety of specific rural subpopulations [4,5,6,7,8,9,10,11,12,13,14], including older rural adults [15,16,17,18,19,20,21]. A few cross-sectional studies have examined differences in physical activity and inactivity between rural and urban populations and found higher inactivity [2,3,22] and lower prevalence of meeting physical activity recommendations [23,24] in rural populations. However, in a cross-sectional study utilizing pedometer data, there were no differences in average steps per day among rural, urban, and suburban participants [25]. Additionally, as part of a cross-sectional analysis on how lifestyle differences impact bone, where rural was synonymous with a farming lifestyle, physical activity was examined in Hutterite non-rural and rural adults, and it was found that rural women were more physically active than their Hutterite and non-rural counterparts and Hutterite and rural men were more physically active than their non-rural counterparts [26]. These findings suggest that living a rural lifestyle, as opposed to simply living in a rural geographic location, plays a role in physical activity behaviors, necessitating further examination of characteristics of populations residing in similar geographic locations. Similar to physical activity, diet patterns have been assessed in a variety of specific rural subpopulations [27,28,29,30,31,32,33,34]. Studies that have compared the diet patterns of rural versus urban adults indicate that rural adults have poorer dietary habits than urban adults [3].

Rural adults may participate in less moderate-to-vigorous physical activity (MVPA) and have poorer dietary behaviors than their urban counterparts, thus contributing to the rural–urban obesity disparity. However, conflicting results among studies about the physical activity and diet behaviors of rural adults warrant further investigation into the role that rural geographic location plays in shaping behaviors versus having a rural occupation or living a rural lifestyle. By examining the behaviors of individuals who reside in similar geographic locations but have different rural occupations and lifestyles, valuable insight into how the rural environment may contribute to the obesity disparity and how rural populations should be defined in future research can be determined. As such, the purpose of this study was to explore and describe both physical activity and diet patterns among three groups of Midwestern adults living in similar geographic locations: (1) non-rural non-Hutterite, (2) rural non-Hutterite, and (3) rural Hutterite. By examining these three groups, occupation-related differences between “rural” and “non-rural” populations can be seen, in addition to potential nuances between rural lifestyles. We anticipated differences in physical activity between both rural groups (occupation-related) and the non-rural group, as well as differences in diet between Hutterites and non-Hutterites (lifestyle-related).

## 2. Materials and Methods

### 2.1. Study Design

The South Dakota Rural Bone Health Study (SDRBHS) is an observational, longitudinal population-based study of 3 healthy groups of adults aged 20–66 years: (1) non-rural non-Hutterite (NH Non-Rural), (2) rural non-Hutterite (NH Rural), and (3) rural Hutterite (Hutterite) [26]. These groups are described in detail elsewhere [26]. Briefly, Hutterite participants had to be living on a Hutterite colony and be of Hutterite descent [26]. Individuals on Hutterite colonies live communally, share goods and resources, and grow and prepare much of their own food. For participants to be considered rural, they had to have spent 75% or more of their life on a working farm while working less than 1040 h/year off the farm [26]. Non-Hutterite rural participants were recruited by calling all individuals who owned land zoned agricultural in 8 counties in eastern South Dakota [26]. Non-rural participants could never have lived on an active working farm, and they were recruited by calling every tenth phone number in local phone books in the same 8 counties and by word of mouth [26]. While there are limitations with phone-based study recruitment, including the exclusion of individuals with unlisted phone numbers, using this method in 2001 to enroll individuals resulted in the desired sample sizes needed to achieve study outcomes. Individuals with uncontrolled type I diabetes, parathyroid disease, or chronic regular use (>6 months) of oral steroids, anticonvulsants, or immunosuppressants were not eligible for the study [26].

The original SDRBHS was 3 years in length, with enrollment beginning in 2001. A baseline visit was conducted in person and information about medical history, diet, and physical activity were collected. Additionally, anthropometric assessments were obtained. In-person visits were conducted again 18 and 36 months after baseline. Diet and physical activity data were also obtained quarterly throughout the 3-year study period to capture seasonal changes in these behaviors. A follow-up to the original study was conducted that consisted only of in-person visits, similar to those described previously, which were conducted every 18 months, at 54, 72, and 90 months after enrollment. No quarterly diet or physical activity data were collected during the follow-up study. The present analysis uses physical activity data from the original study and diet data from the follow-up study. All study protocols and procedures were approved by the South Dakota State University Institutional Review Board.

### 2.2. Assessments

During the original study, the Paffenbarger Physical Activity Questionnaire (PAQ) [35] was administered quarterly to determine self-reported physical activity over the past 7 days and included average number of miles walked per day, usual pace of walking, and average number of flights of stairs walked up each day. A trained interviewer administered the PAQ, and participants reported how much time (in minutes and/or hours) on average they spent per day sleeping, sitting, and in moderate or vigorous activity on both weekdays and weekend days during the past week. Vigorous activity was defined as “your heart rate is elevated and you’re working so hard that you feel out of breath” and examples given included digging in the garden, jogging, snow shoveling, and brisk walking. Moderate activity was defined as being “on your feet and moving around but not working so hard you feel out of breath” and examples given included normal housework, regular walking, yard work, and active games with children. Any remaining time was considered light activity (for a total among all categories of 24 h). Of note, Hutterite adults only have 1 weekend day (Sunday), in the sense that occupational work is not done, whereas the number of weekend days for other NH Non-Rural and NH Rural participants varied.

During the original study, diet data were collected quarterly as 24 h dietary recalls. Originally intended to monitor the intake of key nutrients related to study outcomes, data from these quarterly 24 h dietary recalls are not representative of one’s diet. Examining an individual’s overall diet and food patterns has benefits over examining single nutrients and related outcomes—mostly because people eat foods and meals, not isolated nutrients [36]. As such, diet data from the follow-up study were used in the present analyses. During the follow-up to the original study, a semiquantitative food frequency questionnaire (FFQ) [37] was used to assess average food intake over the previous year. The FFQs were processed by the publisher, and daily servings of individual foods were calculated from responses and summed across food groups, per FFQ documentation [38]. Foods were grouped based on their location within the FFQ (e.g., frozen yogurt under “Dairy”); however, some foods were moved to a more appropriate category and a new category was created to capture “Condiments” (see Supplementary Table S1 for all changes). Of note, this FFQ includes potatoes in the “Starches” food group, not the “Vegetables” food group.

At all in-person study visits where anthropometric data were collected, height without shoes and weight with light clothing were determined with a portable stadiometer (Seca) and digital scale (Seca, Model 770, Hamburg, Germany). Height measurements, recorded to the nearest 0.5 cm, were taken in duplicate by trained staff and repeated if they differed by more than 0.5 cm. Weight was recorded to the nearest 0.1 kg. Body mass index (BMI) was calculated from these measurements.

### 2.3. Subjects

The physical activity analyses include those individuals involved in the original SDRBHS study and comprise 1222 individuals (705 female) from 8 counties in eastern South Dakota who completed data collection at 1 or more of the 3 in-person data collection time points during the original study. Ultimately, for physical activity analyses, data from 327 NH Non-Rural, 339 NH Rural, and 556 Hutterite individuals were analyzed. The diet analyses include those individuals involved in the follow-up to the original SDRBHS study and comprise the 1090 individuals (644 female) who were enrolled in the original study and who completed data collection at 1 or more of the 3 in-person follow-up data collection time points between 2005 and 2012. Ultimately, for diet analyses, data from 274 NH Non-Rural, 301 NH Rural, and 502 Hutterite individuals were analyzed. Hutterite participants were included in both physical activity and diet analyses because although rural, they live communally in a traditional and isolated manner and provide additional information about lifestyle differences (including farming practices and self-sufficiency in food production) that may contribute to rural–urban differences in physical activity and diet patterns.

### 2.4. Statistical Analysis

All analyses were conducted using Stata Statistical Software, Version 14.2 for Windows (StataCorp, 2015; College Station, TX, USA). Demographic variables were compared across groups using one-way ANOVA or chi-square tests and group differences were determined using the Bonferroni test for multiple comparisons. For physical activity analyses, quarterly physical activity data from the original study were averaged over each year of the study to obtain mean values for baseline (quarterly data from 0, 3, 6, and 9 months), 18 months (quarterly data from 15, 18, 21, and 24 months), and 36 months (quarterly data from 27, 30, 33, and 36 months). Mixed models were generated with each physical activity variable as the outcome and included main effects for population group (NH Non-Rural/NH Rural/Hutterite) and time since baseline visit (calculated in months). A random effect for individuals (random intercept) was included to account for longitudinal correlation and a random slope was included to improve model fit. Baseline variables including BMI, age, and season were included as covariates in the models examining physical activity, and age was centered. All analyses were stratified by sex. One subject was excluded from the analysis of stair flights due to an excessive number recorded as a result of exercise (>750 flights/day). Marginal mean values for physical activity (averaged by group from the 3 study visits) were obtained from the model, essentially allowing for cross-sectional comparison. Postestimation Wald tests were used to determine the significance of differences between group mean values.

For diet analyses, FFQ data from each of the 3 follow-up study visits were used. Mixed models were generated for mean servings per day (svg/day) of each food group and included main effects for group (NH Non-Rural/NH Rural/Hutterite), estimated kilocalories per day, season, and time since baseline visit (calculated in months). A random effect for individuals (random intercept) was included to account for longitudinal correlation and a random slope was included to improve model fit. Estimated kilocalories per day were divided by 1000 and baseline age was centered. Analyses were stratified by sex. Adjusted mean daily servings of each food group (averaged by group from the 3 study visits) were obtained from the model, essentially allowing for cross-sectional comparison. Postestimation Wald tests were used to determine the significance of differences between group mean values.

## 3. Results

### 3.1. Anthropometric Assessments and Physical Activity

Detailed demographic information for participants in the original SDRBHS study at the baseline data collection visit are reported in Table 1. Among females at this time point, NH Rural females were oldest, followed by NH Non-Rural and Hutterite females. Hutterite females were the shortest. Among males, Hutterite participants were the youngest and shortest. There were no group differences in weight or BMI among females or males.

Physical activity outcomes are presented in Table 2. In terms of rural vs. non-rural findings, differences included NH Rural females spending more time in moderate activity in general (4.8 ± 0.13 h/day on weekday vs. 3.5 ± 0.11 h/day, *p* < 0.001; and 4.5 ± 0.11 h/day on weekend day vs. 4.1 ± 0.10 h/day, *p* = 0.003) and more time in vigorous activity on weekdays (0.58 ± 0.03 h/day vs. 0.43 ± 0.03 h/day, *p* = < 0.001) compared to NH Non-Rural females, as well as sitting less on weekdays (4.4 ± 0.13 h/day vs. 5.0 ± 0.11 h/day, *p* = < 0.001) and spending less time in light activity on weekdays (6.9 ± 0.15 h/day vs. 7.8 ± 0.13 h/day, *p* < 0.001) and weekend days (7.0 ± 0.14 h/day vs. 7.4 ± 0.13 h/day, *p* = 0.03) than their NH Non-Rural counterparts. Both rural groups (NH Rural and Hutterite) spent more time in moderate activity (4.8 ± 0.13 h/day and 4.7 ± 0.09 h/day vs. 3.5 ± 0.11 h/day, both *p* < 0.001) and vigorous activity (0.58 ± 0.03 h/day and 0.53 ± 0.02 h/day vs. 0.43 ± 0.03 h/day, both *p* < 0.01) and less time sitting (4.4 ± 0.13 h/day and 4.3 ± 0.09 h/day vs. 5.0 ± 0.11 h/day, both *p* < 0.001) on weekdays, and in light activity on weekdays (6.9 ± 0.15 h/day and 5.6 ± 0.10 h/day vs. 7.8 ± 0.13 h/day, both *p* < 0.001) and weekend days (7.0 ± 0.14 h/day and 5.3 ± 0.10 h/day vs. 7.4 ± 0.13 h/day, *p* = 0.03 and *p* < 0.001, respectively) compared to NH Non-Rural females. Among males, differences included NH Rural males spending more time in moderate activity in general (4.9 ± 0.13 h/day on weekday vs. 3.0 ± 0.16 h/day, *p* < 0.001; and 4.1 ± 0.11 h/day on weekend day vs. 3.6 ± 0.13 h/day, *p* = 0.002) and more time in vigorous activity on weekdays (0.82 ± 0.05 h/day vs. 0.63 ± 0.06 h/day, *p* = 0.01) compared to NH Non-Rural males, as well as spending less time sitting on weekdays (4.1 ± 0.13 h/day vs. 6.0 ± 0.15 h/day, *p* < 0.001). Both rural groups (NH Rural and Hutterite) spent more time in moderate activity on weekdays (4.9 ± 0.13 h/day and 6.1 ± 0.12 h/day vs. 3.0 ± 0.16 h/day, both *p* < 0.001) and less time sitting on weekdays (4.1 ± 0.13 h/day and 3.4 ± 0.12 h/day vs. 6.0 ± 0.15 h/day, both *p* < 0.001) compared to NH Non-Rural males.

Between Hutterite and NH Rural females, time spent in moderate activity (4.7 ± 0.09 h/day and 4.8 ± 0.13 h/day, *p* = 0.54) and vigorous activity (0.53 ± 0.02 h/day and 0.58 ± 0.03 h/day, *p* = 0.20) on weekdays did not differ; however, Hutterite females, on average, spent less time in moderate activity (2.6 ± 0.08 h/day vs. 4.5 ± 0.11 h/day, *p* < 0.001) and vigorous activity (0.18 ± 0.02 h/day vs. 0.46 ± 0.02 h/day, *p* < 0.001) on weekend days compared to their NH Rural counterparts. Hutterite females also spent significantly less time in light activity on weekdays (5.6 ± 0.10 h/day vs. 6.9 ± 0.15 h/day, *p* < 0.001) and weekend days (5.3 ± 0.10 h/day vs. 7.0 ± 0.14 h/day, *p* < 0.001). Additionally, Hutterite females walked more miles per day (2.7 ± 0.06 vs. 2.1 ± 0.08, *p* < 0.001) and climbed more stair flights per day (10.5 ± 0.30 vs. 7.2 ± 0.43, *p* < 0.001) than their NH Rural counterparts, and spent more time sleeping on weekdays (8.8 ± 0.04 h/day vs. 7.4 ± 0.06 h/day, *p* < 0.001) and weekend days (10.2 ± 0.05 h/day vs. 7.8 ± 0.07 h/day, *p* < 0.001) and sitting on weekend days (5.8 ± 0.07 h/day vs. 4.2 ± 0.10 h/day, *p* < 0.001). Hutterite males, on average, spent more time in moderate activity on weekdays (6.1 ± 0.12 h/day vs. 4.9 ± 0.13 h/day, *p* < 0.001) and less time in moderate activity (2.1 ± 0.10 h/day vs. 4.1 ± 0.11 h/day, *p* < 0.001) and vigorous activity (0.15 ± 0.04 h/day vs. 0.74 ± 0.04 h/day, *p* < 0.001) on weekend days compared to their NH Rural counterparts. They also walked more miles per day (3.1 ± 0.09 vs. 1.9 ± 0.09, *p* < 0.001) and climbed more stair flights per day (8.8 ± 0.37 vs. 6.9 ± 0.40, *p* < 0.001) than their NH Rural counterparts. Additionally, compared to NH Rural males, Hutterite participants spent more time sleeping and less time in light activity on weekdays (8.4 ± 0.05 h/day vs. 7.3 ± 0.05 h/day, *p* < 0.001; and 5.4 ± 0.13 h/day vs. 6.9 ± 0.13 h/day, *p* < 0.001) and weekend days (10.2 ± 0.06 h/day vs. 7.7 ± 0.06 h/day, *p* < 0.001; and 6.2 ± 0.13 h/day vs. 7.1 ± 0.14 h/day, *p* < 0.001), and more time sitting on weekend days (5.4 ± 0.10 h/day vs. 4.4 ± 0.10 h/day, *p* < 0.001).

### 3.2. Anthropometric Assessments and Diet

Detailed demographic information for participants in the SDRBHS follow-up study at the baseline diet collection visit (54 months postenrollment) are also reported in Table 1. At this time point, Hutterite females were younger and shorter than NH Non-Rural and NH Rural participants. Hutterite males were also younger than NH Non-Rural and NH Rural participants and were shorter than their NH Non-Rural counterparts. Both female and male Hutterite participants had higher mean BMI than NH Non-Rural participants.

Diet outcomes are presented in Table 3. In terms of rural vs. non-rural findings, differences included NH Rural females consuming more total calories per day than NH Non-Rural females (2039 ± 54 vs. 1833 ± 50, *p* = 0.005), but fewer calorie-containing beverages (0.50 ± 0.05 svg/day vs. 0.68 ± 0.04 svg/day, *p* = 0.005). While Hutterite females consumed fewer calories per day than NH Rural females (1830 ± 37 vs. 2039 ± 54, *p* = 0.002), both rural groups consumed fewer calorie-containing beverages than NH Non-Rural females (0.44 ± 0.03 svg/day and 0.50 ± 0.05 svg/day vs. 0.68 ± 0.04 svg/day, *p* = < 0.001 and *p* = 0.005, respectively). Differences between NH Rural and NH Non-Rural participants also included NH Rural males consuming more total calories per day than NH Non-Rural males (2432 ± 52 vs. 2202 ± 61, *p* = 0.004), as well as greater amounts of sweets/miscellaneous foods (1.6 ± 0.07 svg/day vs. 1.3 ± 0.09 svg/day, *p* = 0.03). While Hutterite males consumed fewer calories per day than NH Non-Rural males (2033 ± 47 vs. 2202 ± 61, *p* = 0.03), both rural groups consumed more sweets/miscellaneous foods than NH Non-Rural males (1.7 ± 0.07 svg/day and 1.6 ± 0.07 svg/day vs. 1.3 ± 0.09 svg/day, *p* = 0.001 and *p* = 0.03, respectively).

In terms of Hutterite vs. NH Rural findings, Hutterite females, on average, consumed less dairy (1.8 ± 0.06 svg/day vs. 2.2 ± 0.08 svg/day, *p* < 0.001) and starches (2.9 ± 0.06 svg/day vs. 3.5 ± 0.08 svg/day, *p* < 0.001) and more fruits (2.2 ± 0.06 svg/day vs. 1.7 ± 0.10 svg/day, *p* < 0.001) and vegetables (3.6 ± 0.08 svg/day vs. 2.7 ± 0.12 svg/day, *p* < 0.001) than their NH Rural counterparts. They also consumed significantly more sweets/miscellaneous foods (1.4 ± 0.04 svg/day vs. 1.1 ± 0.06 svg/day, *p* < 0.001) and condiments (4.1 ± 0.10 svg/day vs. 3.5 ± 0.15 svg/day, *p* < 0.001). Hutterite males, on average, consumed fewer calories per day (2033 ± 47 vs. 2432 ± 52, *p* < 0.001) and less dairy (2.0 ± 0.08 svg/day vs. 2.4 ± 0.09 svg/day, *p* = 0.005) and starches (3.2 ± 0.09 svg/day vs. 4.1 ± 0.10 svg/day, *p* < 0.001) than their NH Rural counterparts, but consumed more vegetables (3.0 ± 0.10 svg/day vs. 2.0 ± 0.11 svg/day, *p* < 0.001) and low-calorie beverages (6.1 ± 0.16 svg/day vs. 4.8 ± 0.18 svg/day, *p* < 0.001).

## 4. Discussion

A disparity in obesity prevalence exists between rural and urban populations. It is unclear how physical activity and diet patterns may be contributing to this disparity, and little work exists exploring how physical activity and diet behaviors are shaped by rural occupation or lifestyle, as opposed to simply living in a rural area (as defined by geography or population size). As such, the purpose of this study was to explore and describe physical activity and diet behaviors in rural versus non-rural Midwestern adults, with “rural” indicating living and working on an active farm. To determine if and how behaviors could be attributed to a rural lifestyle, two groups of rural adults (Hutterite and non-Hutterite) were assessed, in addition to one group of NH Non-Rural adults.

### 4.1. Physical Activity

#### 4.1.1. Rural vs. Non-Rural

Large cross-sectional studies indicate that rural populations have higher levels of physical inactivity than urban populations [2,22,23]. This was confirmed by accelerometer data collected as part of NHANES; however, it differs from self-reported physical activity obtained within the same study [39]. Contrary to these findings, and similar to cross-sectional findings previously reported in this study population [26], the present study indicates that among both females and males, time spent in moderate activity on weekdays was higher in both rural groups compared to the NH Non-Rural group. Likewise, for both females and males, both rural groups spent fewer hours sitting on weekdays than the NH Non-Rural group. Among females, vigorous activity on weekdays was also higher in rural groups vs. NH Non-Rural. Classifying individuals based on whether or not they farm as an occupation provides additional insight into why individuals living a rural lifestyle would report more MVPA than NH Non-Rural. In previous studies, “rural” was defined using either rural-urban commuting area codes, Rural-Urban Continuum codes, or metropolitan statistical area. In the present study, given that all study participants came from the same geographic locations, contrary findings are likely due to differences in physical activity demands related to living and working as part of a rural lifestyle. For example, living a rural lifestyle may involve walking to and from areas on a farm to check on livestock or crops, while NH Non-Rural individuals may be more likely to work in an office setting or at a desk that requires less physical labor. Future work should not only compare measured versus reported physical activity among those living a rural lifestyle versus those who are not, but also pair measured assessments with self-reported logs in order to capture information about physical activity type (including recreational activity, activities of daily living, and workplace activity), in addition to intensity and duration. Use of a self-reported physical activity assessment tool such as the Global Physical Activity Questionnaire (GPAQ) would also be appropriate, as it captures physical activity in multiple domains, including occupational and recreational, addressing potential lifestyle differences within rural populations [40].

The latest guidelines for physical activity in adults recommend at least 150 min (2.5 h) per week of moderate-intensity aerobic activity [41]. In the present study, NH Non-Rural males spent the least amount of time in moderate activity on weekdays, averaging around 3 h per day—an amount higher than recommendations for accumulated moderate activity over the course of a week. This discrepancy is likely due to assessment methodology and the definition of MVPA. The PAQ used in the present study classified moderate activity essentially as being on your feet and moving around. Although this specific assessment has been shown to correlate with actual physical activity [42] and with miles walked in Hutterite populations [43], “moderate” activity referred to in recommendations now is more reflective of a level needed to elicit health benefits. The 2008 Physical Activity Guidelines for Americans specify that moderate activity should be aerobic, meaning “physical activities in which people move their large muscles in a rhythmic manner for a sustained period,” and should occur in bouts of at least 10 min [41]. This type of activity increases heart rate, and examples of moderate intensity include brisk walking, water aerobics, and doubles tennis [41]. The key difference between that definition and the one used in the present study is likely the inclusion of “rhythmic” and “sustained” in the former but not the latter. Although participants reported their moderate activity based on the definition they were given, it may not have been moderate “aerobic” activity and may not have been sustained for more than 10 min at a time, therefore inflating overall reports of moderate physical activity compared to other studies. Despite these limitations with interpretation, differences in physical activity levels were seen between female and male rural and non-rural population groups.

#### 4.1.2. Hutterite vs. NH Rural

There are some differences in physical activity outcomes between Hutterite and NH Rural adults that highlight the nuances of living a rural lifestyle and warrant further investigation. For example, despite both groups being rural and living and working on active farms, male Hutterite participants spent more time in moderate activity on weekdays than NH Rural males. Additionally, Hutterite males and females walked more miles on average and males sat less on weekdays. It is unclear why physical activity levels differ on weekdays between these two rural groups, especially when Hutterite farming operations are highly mechanized. We speculate that those in the NH rural group have access to motorized vehicles for transportation to and from farm duties or chores, and that Hutterite participants lack that access and must walk from location to location. Additionally, Hutterite females must walk to a central location to do chores like laundry and meal preparation, as these do not take place within individual homes. Future research capturing types of physical activity and how time is spent, in addition to quantity of physical activity, would further inform these findings.

Hutterite participants spent less time in moderate and vigorous activity and more time sleeping and sitting on weekend days than non-Hutterite participants. Differences in weekend day activity may be attributed to Hutterite participants having only Sunday as a weekend day, during which they spend a significant amount of time in church (up to 2–4 h), and the tradition of taking a long afternoon nap, characteristics of living a more “traditional” lifestyle where one works for six days a week and rests (including naps) on the seventh day. These findings highlight how a rural occupation may influence physical activity levels, and how other lifestyle differences further add to variances in physical activity within rural populations. It is clear that more than geographic location is needed to understand and quantify differences in physical activity between rural and non-rural population groups.

### 4.2. Diet

#### 4.2.1. Rural vs. Non-Rural

Large cross-sectional studies indicate that rural populations are less likely to consume recommended amounts of fruits and vegetables compared to non-rural adults [33], and in general have poorer diets than their urban counterparts [3]. In the present study, while frequency of meeting recommendations was not specifically determined, NH Non-Rural females consumed significantly more calorie-containing beverages than their rural counterparts, and rural males consumed significantly more sweets and miscellaneous foods than their NH Non-Rural counterparts. Across all three groups and among both females and males, NH Rural individuals consumed the most calories. While both rural and non-rural diets could be improved to be more in line with recommendations, one was not found to be systematically worse than the other.

Overall, very few dietary outcomes differed between the two non-Hutterite population groups, despite being rural and non-rural. Given that the present study classified individuals as rural or non-rural based on whether they were actively farming, it may be the case that a rural individual lives and works on a farm, but another individual in the family works off the farm (i.e., in town) and has access to locations where food is sold or prepared. If not, perhaps regular trips are made to town for food shopping and other activities. Transportation, food processing, modes of shopping, and home food storage likely all contribute to both NH populations having the same access to, and ultimately consumption of, most food groups.

#### 4.2.2. Hutterite vs. NH Rural

A number of differences in dietary patterns were seen between the two rural population groups, indicating that rural lifestyle shapes eating behaviors. For both females and males, Hutterite participants consumed less dairy, more vegetables, and fewer starches than NH Rural individuals. Additionally, Hutterite females consumed more fruit, sweets/miscellaneous foods, and condiments than NH Rural females, while Hutterite males consumed more low-calorie beverages than NH Rural males. As the majority of differences in diet outcomes are between Hutterite participants and the other two population groups, diet appears to be more influenced by type of rural lifestyle than by rural occupation or geographic location. Fluid milk is less commonly consumed among the Hutterite Brethren, which may explain the differences in intake between Hutterite and both NH Rural and NH Non-Rural individuals. Hutterites produce and process much of their own food, including growing and canning fruits and vegetables, making yogurt, and baking breads and desserts. Meals are prepared in a central kitchen and served family-style in a central dining room. These again may be aspects of a more “traditional” rural lifestyle, where one is self-sufficient when it comes to food, and where grocery and restaurant options are not readily accessible.

### 4.3. Limitations

There are certain limitations to this study that should be taken into consideration. Participants were asked to subjectively recall their levels of physical activity over the past week, which may have led to over- or underestimation. Additionally, the definition of “moderate” activity used in this study may differ from others, limiting the comparison of results with other studies. With regard to diet, participants were asked to indicate average intake of foods over the past year—this can be difficult when trying to average one’s intake of foods that are eaten only seasonally. Moreover, serving size or its appropriate equivalent for recommended intake was not always captured. For example, the FFQ ascertains how often one hamburger patty is eaten, but does not specify the size of the patty; “servings” of cottage cheese are captured in half-cup increments, but 2 cups are required to be equivalent to an actual 1 cup serving of dairy per current dietary guidelines (USDA’s MyPlate). These issues make it difficult to quantify servings of foods. Despite these limitations, we were able to characterize patterns of physical activity and dietary intake in Hutterite, NH Rural, and NH Non-Rural females and males, providing insight into why an obesity disparity may exist between rural and urban populations and how living a rural lifestyle may shape contributing behaviors. Additional strengths of this study include the use of multiple physical activity recalls over a period of three years spanning all seasons for analysis. Finally, rural was defined for individuals as actually living a rural lifestyle, not just based on geography, population, or zoning regulations.

## 5. Conclusions

In this study, where rural was defined based on an individual’s occupation and lifestyle as opposed to simply his or her geographic location, differences between rural and non-rural populations were seen in both self-reported physical activity and dietary patterns. Aspects of a rural occupation and lifestyle contribute to differences in physical activity patterns, while traditional rural lifestyle practices contribute to differences in diet. To build upon these findings and complement the existing literature in this area, assessment of measured physical activity, such as with accelerometers, should be used in future studies, in addition to logs indicating the types of activities being engaged in or self-reported physical activity assessments that capture activity in multiple domains. Similarly, diet data should be obtained using multiple 24 h dietary recalls, ideally collected systematically, across seasons, and on both weekday and weekend days, while also capturing where foods are obtained. Having physical activity and diet data collected in this way, and capturing information about other factors within the home and community environments associated with weight status, would greatly enhance our understanding of how rural geography, occupation, and lifestyle contribute to the rural–urban obesity disparity.

## Figures and Tables

**Table 1:** Unadjusted baseline demographics of original and follow-up cohorts (mean ± standard error).

	NH Non-Rural	NH Rural	Hutterite	*p*-Value ^1^
**Baseline (month 0)**				
Females	*n* = 199	*n* = 163	*n* = 343	
Season at enrollment (F/Sp/Su/W)	72/29/24/74	31/44/45/43	48/70/137/88	<0.001
Weekdays (no.) ^2^	5.0 (3–6) ^ab^	5.4 (5–6) ^ac^	6.0 (5–6) ^bc^	<0.001
Age (years)	42.8 ± 0.44 ^ab^	48.6 ± 0.63 ^ac^	39.7 ± 0.39 ^bc^	<0.001
Height (cm)	163.8 ± 0.27 ^a^	164.2 ± 0.28 ^b^	161.8 ± 0.17 ^ab^	<0.001
Weight (kg)	73.4 ± 0.70	74.3 ± 0.67	74.8 ± 0.52	0.98
BMI (kg/m^2^)	27.3 ± 0.24	27.6 ± 0.26	28.6 ± 0.20	0.32
Normal weight/overweight or obese	81/118	59/104	124/219	0.53
Males	*n* = 128	*n* = 176	*n* = 213	
Season at enrollment (F/Sp/Su/W)	41/15/11/61	46/39/60/31	31/48/92/42	<0.001
Weekdays (no.) ^2^	5.0 (3–6) ^ab^	5.6 (4–6) ^ac^	6.0 (6–6) ^bc^	<0.001
Age (years)	43.6 ± 0.61 ^a^	46.2 ± 0.59 ^b^	38.7 ± 0.47 ^ab^	<0.001
Height (cm)	178.9 ± 0.38 ^a^	178.1 ± 0.33 ^b^	176.5 ± 0.23 ^ab^	<0.01
Weight (kg)	91.5 ± 0.93	94.5 ± 0.72	92.3 ± 0.65	0.17
BMI (kg/m^2^)	28.5 ± 0.28	29.8 ± 0.21	29.6 ± 0.19	0.12
Normal weight/overweight or obese	33/95	30/146	38/175	0.12
**Follow-up (month 54)**				
Females	*n* = 168	*n* = 151	*n* = 320	
Age (years) ^2^	47.0 ± 0.47 ^ab^	52.8 ± 0.62 ^ac^	43.4 ± 0.40 ^bc^	<0.001
Height (cm)	163.4 ± 0.29 ^a^	163.1 ± 0.30 ^b^	161.2 ± 0.18 ^ab^	<0.001
Weight (kg)	74.4 ± 0.76	75.0 ± 0.75	77.7 ± 0.54	0.14
BMI (kg/m^2^)	27.8 ± 0.27 ^a^	28.2 ± 0.28	29.9 ± 0.22 ^a^	<0.01
Normal weight/overweight or obese	46/122	42/109	74/246	0.43
Male	*n* = 106	*n* = 150	*n* = 182	
Age (years) ^2^	48.2 ± 0.64 ^a^	50.9 ± 0.58 ^b^	41.9 ± 0.49 ^ab^	<0.001
Height (cm)	178.2 ± 0.45 ^a^	177.7 ± 0.39	175.9 ± 0.25 ^a^	<0.01
Weight (kg)	91.4 ± 1.1	95.3 ± 0.76	94.0 ± 0.71	0.24
BMI (kg/m^2^)	28.8 ± 0.33 ^a^	30.2 ± 0.23	30.4 ± 0.21 ^a^	0.02
Normal weight/overweight or obese	23/83	13/137	16/166	0.002

F, fall; Sp, spring; Su, summer; W, winter; BMI, body mass index; NH, non-Hutterite. ^1^ Overall differences among groups examined using one-way ANOVA or chi-square; means with similar superscripts are different at *p* ≤ 0.05 (Bonferroni test for multiple comparisons). ^2^ Mean (range).

**Table 2:** Marginal means (±SE) and *p*-values for physical activity (per week and weekend day), stratified by sex. ^1,3,4^

	Miles Walked ^2^	Stair Flights ^2^	Sleep Weekday	Sleep Weekend	Sit Weekday	Sit Weekend	Light Weekday	Light Weekend	Moderate Weekday	Moderate Weekend	Vigorous Weekday	Vigorous Weekend
Females												
NH Non-Rural	2.1 ± 0.07 ^a^	8.2 ± 0.38 ^a^	7.3 ± 0.06 ^a^	7.8 ± 0.06 ^a^	5.0 ± 0.11 ^ab^	4.3 ± 0.09 ^a^	7.8 ± 0.13 ^ab^	7.4 ± 0.13 ^ab^	3.5 ± 0.11 ^ab^	4.1 ± 0.10 ^ab^	0.43 ± 0.03 ^ab^	0.47 ± 0.002 ^a^
NH Rural	2.1 ± 0.08 ^b^	7.2 ± 0.43 ^b^	7.4 ± 0.06 ^b^	7.8 ± 0.07 ^b^	4.4 ± 0.13 ^a^	4.2 ± 0.10 ^b^	6.9 ± 0.15 ^ac^	7.0 ± 0.14 ^ac^	4.8 ± 0.13 ^a^	4.5 ± 0.11 ^ac^	0.58 ± 0.03 ^a^	0.46 ± 0.02 ^b^
Hutterite	2.7 ± 0.06 ^ab^	10.5 ± 0.30 ^ab^	8.8 ± 0.04 ^ab^	10.2 ± 0.05 ^ab^	4.3 ± 0.09 ^b^	5.8 ± 0.07 ^ab^	5.6 ± 0.10 ^bc^	5.3 ± 0.10 ^bc^	4.7 ± 0.09 ^b^	2.6 ± 0.08 ^bc^	0.53 ± 0.02 ^b^	0.18 ± 0.02 ^ab^
Male												
NH Non-Rural	2.2 ± 0.11 ^a^	9.5 ± 0.47 ^a^	7.1 ± 0.06 ^a^	7.6 ± 0.07 ^a^	6.0 ± 0.15 ^ab^	4.7 ± 0.12 ^a^	7.3 ± 0.16 ^a^	7.4 ± 0.16 ^a^	3.0 ± 0.16 ^ab^	3.6 ± 0.13 ^ab^	0.63 ± 0.06 ^a^	0.74 ± 0.05 ^a^
NH Rural	1.9 ± 0.09 ^b^	6.9 ± 0.40 ^ab^	7.3 ± 0.05 ^b^	7.7 ± 0.06 ^b^	4.1 ± 0.13 ^ac^	4.4 ± 0.10 ^b^	6.9 ± 0.14 ^b^	7.1 ± 0.14 ^b^	4.9 ± 0.13 ^ac^	4.1 ± 0.11 ^ac^	0.82 ± 0.05 ^a^	0.74 ± 0.04 ^b^
Hutterite	3.1 ± 0.09 ^ab^	8.8 ± 0.37 ^b^	8.4 ± 0.05 ^ab^	10.2 ± 0.06 ^ab^	3.4 ± 0.12 ^bc^	5.4 ± 0.10 ^ab^	5.4 ± 0.13 ^ab^	6.2 ± 0.13 ^ab^	6.1 ± 0.12 ^bc^	2.1 ± 0.10 ^bc^	0.74 ± 0.05	0.15 ± 0.04 ^ab^

^1^ Similar superscripts (^a,b,c^) indicate significant difference between groups within sex and outcome variable at *p* ≤ 0.05. ^2^ Values for miles and stairs are per day. ^3^ Values for sleep, sit, light, moderate, and vigorous are in hours per day. ^4^ Analyses controlled for time (months since baseline visit), baseline age (centered), body mass index, and season.

**Table 3:** Marginal means (±SE) of servings by food groups. ^1,2^

	Total Calories	Dairy	Fruits	Vegetables	Meat	Starches	Lo-Cal Beverages	Caloric Beverages	Sweets & Misc	Condiments
Females										
NH Non-Rural	1833 ± 50 ^a^	2.1 ± 0.08 ^a^	1.8 ± 0.09 ^a^	2.8 ± 0.11 ^a^	2.6 ± 0.06	3.4 ± 0.08 ^a^	5.8 ± 0.18 ^a^	0.68 ± 0.04 ^ab^	1.2 ± 0.06 ^a^	3.8 ± 0.14
NH Rural	2039 ± 54 ^ab^	2.2 ± 0.08 ^b^	1.7 ± 0.10 ^b^	2.7 ± 0.12 ^b^	2.8 ± 0.06 ^a^	3.5 ± 0.08 ^b^	5.9 ± 0.20	0.50 ± 0.05 ^a^	1.1 ± 0.06 ^b^	3.5 ± 0.15 ^a^
Hutterite	1830 ± 37 ^b^	1.8 ± 0.06 ^ab^	2.2 ± 0.06 ^ab^	3.6 ± 0.08 ^ab^	2.6 ± 0.04 ^a^	2.9 ± 0.06 ^ab^	6.4 ± 0.13 ^a^	0.44 ± 0.03 ^b^	1.4 ± 0.04 ^ab^	4.1 ± 0.10 ^a^
Male										
NH Non-Rural	2202 ± 61 ^ab^	2.3 ± 0.11 ^a^	1.6 ± 0.10	2.4 ± 0.13 ^a^	3.1 ± 0.09 ^a^	4.1 ± 0.11 ^a^	5.1 ± 0.21 ^a^	1.5 ± 0.09	1.3 ± 0.09 ^ab^	3.9 ± 0.16
NH Rural	2432 ± 52 ^ac^	2.4 ± 0.09 ^b^	1.7 ± 0.09	2.0 ± 0.11 ^b^	3.2 ± 0.08	4.1 ± 0.10 ^b^	4.8 ± 0.18 ^b^	1.3 ± 0.08	1.6 ± 0.07 ^a^	3.5 ± 0.14
Hutterite	2033 ± 47 ^bc^	2.0 ± 0.08 ^ab^	1.8 ± 0.08	3.0 ± 0.10 ^ab^	3.4 ± 0.07 ^a^	3.2 ± 0.09 ^ab^	6.1 ± 0.16 ^ab^	1.3 ± 0.07	1.7 ± 0.07 ^b^	3.5 ± 0.12

^1^ Similar superscripts (^a,b,c^) indicate significant difference between groups within sex and outcome variable at *p* ≤ 0.05. ^2^ Analyses controlled for kilocalories (except for calories as outcome), time (months since 54-month follow-up visit), baseline age (centered), and season.

## Supplementary Materials

The following are available online at http://www.mdpi.com/2072-6643/10/11/1601/s1: Table S1: Changes made to food categories for analysis.
