# Supplementary material for: Differences in Physical Activity and Diet Patterns between Non-Rural and Rural Adults
Source: Nutrients. 2018 Nov 1;10(11):1601. doi: 10.3390/nu10111601 (PMC6267585; doi:10.3390/nu10111601)
Supplement: Supplementary file 1 [file nutrients-10-01601-s001.pdf]

Table S1. Changes made to food categories for analysis.

Food	Original Category on FFQ	Category Used for Analysis
Cream, e.g., coffee, whipped or sour cream	Dairy Foods	Condiments
Non-dairy coffee whitener	Dairy Foods	Condiments
Spreads added to food or bread; exclude use in cooking	Dairy Foods	Condiments
Cream cheese	Dairy Foods	Condiments
Tomatoes (slices)	Vegetables	Condiments
Peppers: green, yellow or red (slices)	Vegetables	Condiments
Onions as a garnish or in a salad	Vegetables	Condiments
Jams, jellies, preserves, syrup, or honey	Sweets, Baked Goods, Miscellaneous	Condiments
Oat bran, added to food	Sweets, Baked Goods, Miscellaneous	Condiments
Other bran (wheat, etc.), added to food	Sweets, Baked Goods, Miscellaneous	Condiments
Ketchup or red chili sauce	Sweets, Baked Goods, Miscellaneous	Condiments
Splenda	Sweets, Baked Goods, Miscellaneous	Condiments
Other artificial sweetener	Sweets, Baked Goods, Miscellaneous	Condiments
Olive oil added to food or bread	Sweets, Baked Goods, Miscellaneous	Condiments
Low-fat or fat-free mayonnaise	Sweets, Baked Goods, Miscellaneous	Condiments
Regular mayonnaise	Sweets, Baked Goods, Miscellaneous	Condiments
Salad dressing	Sweets, Baked Goods, Miscellaneous	Condiments
Peanut butter	Sweets, Baked Goods, Miscellaneous	Meat
Peanuts	Sweets, Baked Goods, Miscellaneous	Meat
Walnuts	Sweets, Baked Goods, Miscellaneous	Meat
Other nuts	Sweets, Baked Goods, Miscellaneous	Meat
Popcorn	Sweets, Baked Goods, Miscellaneous	Starches
Pretzels	Sweets, Baked Goods, Miscellaneous	Starches
